# Application of Organoids in Carcinogenesis Modeling and Tumor Vaccination

**DOI:** 10.3389/fonc.2022.855996

**Published:** 2022-03-17

**Authors:** Zeyu Wang, Shasha Zhao, Xiaolin Lin, Guanglong Chen, Jiawei Kang, Zhongping Ma, Yiming Wang, Zhi Li, Xiuying Xiao, Aina He, Dongxi Xiang

**Affiliations:** ^1^ Department of Gastrointestinal Surgery, Ren Ji Hospital, School of Medicine, Shanghai Jiao Tong University, Shanghai, China; ^2^ State Key Laboratory of Oncogenes and Related Genes, the Renji Hospital Affiliated to Shanghai Jiaotong University School of Medicine, Shanghai, China; ^3^ Department of Oncology, Ren Ji Hospital, Shanghai Jiao Tong University School of Medicine, Shanghai, China; ^4^ Department of General Surgery, Zhengzhou University, Affiliated Cancer Hospital (Henan Cancer Hospital), Zhengzhou, China; ^5^ College of Fisheries and Life Science, Shanghai Ocean University, Shanghai, China; ^6^ Shanghai OneTar Biomedicine, Shanghai, China; ^7^ Department of Oncology, The Sixth People’s Hospital Affiliated to Shanghai Jiaotong University School of Medicine, Shanghai, China; ^8^ State Key Laboratory of Oncogenes and Related Genes, Department of Biliary-Pancreatic Surgery, The Renji Hospital Affiliated to Shanghai Jiaotong University School of Medicine, Shanghai, China

**Keywords:** organoids, tumor-initiating cell, tumorigenesis, precision medicine, tumor vaccine

## Abstract

Organoids well recapitulate organ-specific functions from their tissue of origin and remain fundamental aspects of organogenesis. Organoids are widely applied in biomedical research, drug discovery, and regenerative medicine. There are various cultivated organoid systems induced by adult stem cells and pluripotent stem cells, or directly derived from primary tissues. Researchers have drawn inspiration by combination of organoid technology and tissue engineering to produce organoids with more physiological relevance and suitable for translational medicine. This review describes the value of applying organoids for tumorigenesis modeling and tumor vaccination. We summarize the application of organoids in tumor precision medicine. Extant challenges that need to be conquered to make this technology be more feasible and precise are discussed.

## Introduction

**Figure 1:**
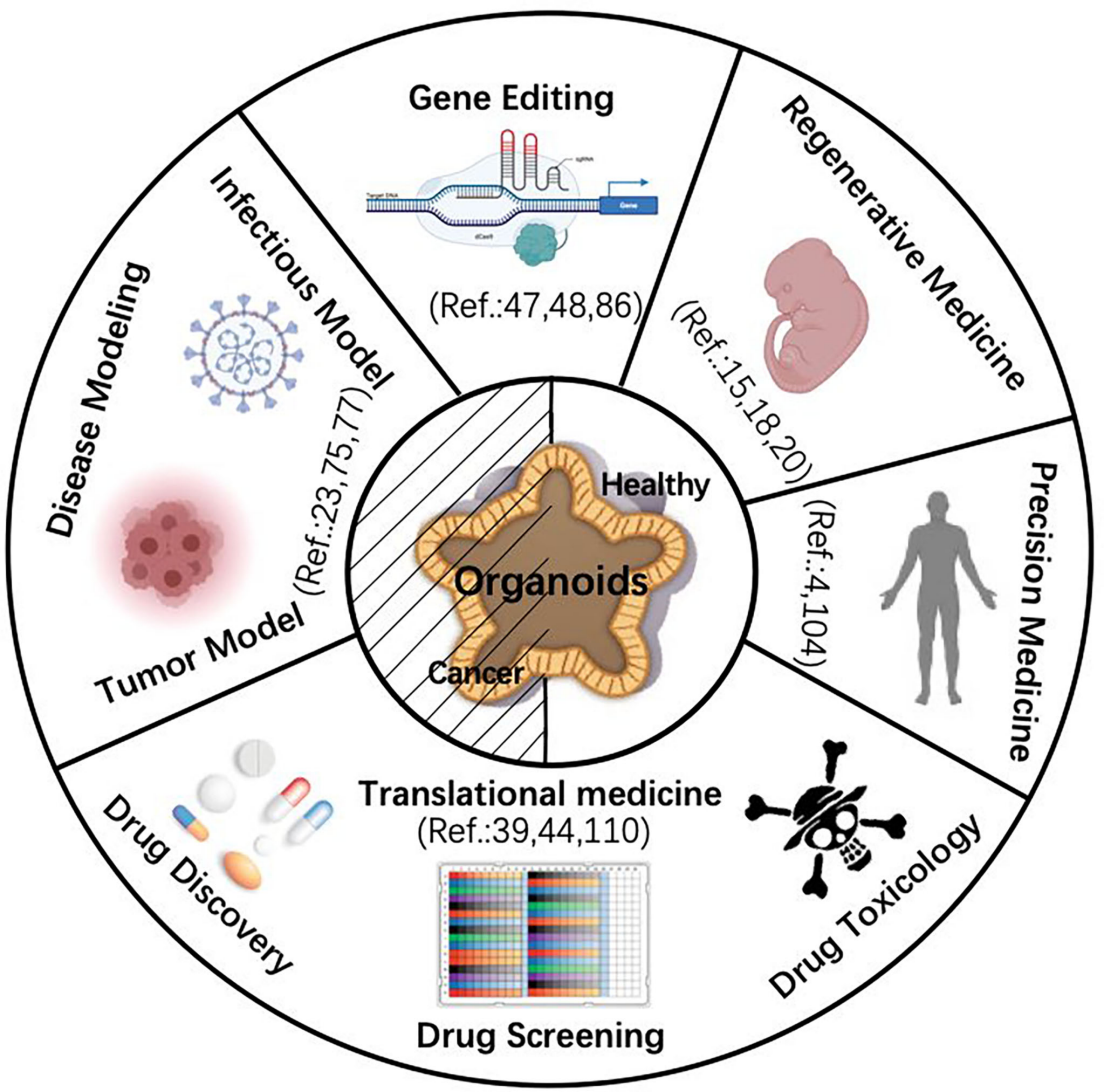
Schematic diagram of organoid application. The center of the diagram shows that both normal and cancer organoids share the five application fields indicated by the peripheral parts. [Note: e.g., Ref. 4 refers to reference (4)]. Images are adapted from Servier Medical Art (https://smart.servier.com).

Organoids are three-dimensional cell complexes with a particular spatial structure cultured *in vitro* (1, 2). It is amplified and maintains certain structural and functional features of their source tissue (3). Organoids develop from stem-like cells or initiating cells including embryonic stem cells (ESCs) (4, 5), adult stem cells (ASCs) (6), induced pluripotent stem cells (iPSCs), and progenitor cells (7–9). ESCs are cells selected from the intraembryonic cell mass or obtained by inhibiting primordial germ cells *in vitro*, which has the ability of multidirectional differentiation (10, 11). ASCs are undifferentiated cells existing in various differentiated tissues that are responsible for repair and regeneration after tissue injury (5). Progenitor cells can repair and regenerate following tissue damage (12). In this review, we demonstrate organoid platforms derived from primary tissues, ASCs or iPSCs, summarize the organoid bioengineering advancement, and describe the possibility of applying organoids for carcinogenesis modeling (**Figure 1**). Current challenges about the broad application of organoids are also discussed.

## Organoid Culture From Primary Tissues

### Organoid Culture From Stem Cells

Organoid culture became a major technological advance in 2009. Hans Clevers reconstructed a suitable niche better for maintaining intestinal stem cells *in vitro* (13). This system contains a key conditioned medium supplemented with multiple growth factors including Wnt agonists R-spondin-1 or -3, Wnt3a, epidermal growth factor (EGF), and noggin. Mouse primary intestine cells were embedded into hydrogel [i.e., extracellular matrix (ECM)] followed by gut organoid formation with villus structure owing to the self-renewal capacity of intestinal stem cells, which has pioneered the field of organoid cancer biology (13). The system guaranteed continuous proliferation of organoids, not only ensured stability and purification of mouse genome but also had an advantage of amplification (14). Research fellows have cultivated diverse organoid systems derived from tissues with epithelial origination, such as bladder, colon, rectum, endometrium, fallopian tube, kidney, liver, lung, esophagus, oral mucosa, pancreas, prostate, salivary gland, skin epidermis, stomach, and taste buds (15–25). Researchers have also generated organoids from normal cells in the urinary tract and bronchial lavage, rather than parenchymatous organs (26, 27). Organoids derived from ASCs maintain phenotypic and genetic stability, better reflecting their primary tissue genome (28, 29). Genomic mutation in organoids can be investigated by immunohistochemistry staining, whole-exome sequencing (WES), and RNA sequencing (30). Organoid is driven by the inherent ability of stem cells themselves (13). Their self-assembly ability allows organoids to produce functional mature “organs” by precise spatial and temporal order (31, 32). Similarly, organoids run self-assembly processes *in vitro* by changing cytokine constituents of culture media, simulating organoid differentiation and maturation (14, 33, 34). Gabriel et al. (35) observed that brain organoids assembled optic vesicles, including primitive corneal epithelial and lens-like cells. This study confirmed that the self-assembly process was carried out *via* a multistep process in the early stage of organoid genesis.

Organoid culture varies from different tissues of origin; several organs require more efforts to establish a stable condition (i.e., heart and immune organs) (36–38). It has been studied to generate complex and highly structured cardiac organs by embedding human iPSCs into matrix glue, regulating small molecule directional cardiac differentiation through the biphasic Wnt pathway (39). The lacrimal secretion of neurotransmitters by lacrimal gland organoids was verified by orthotopic transplantation in mice (40).

### Organoid Bioengineering

**Figure 2:**
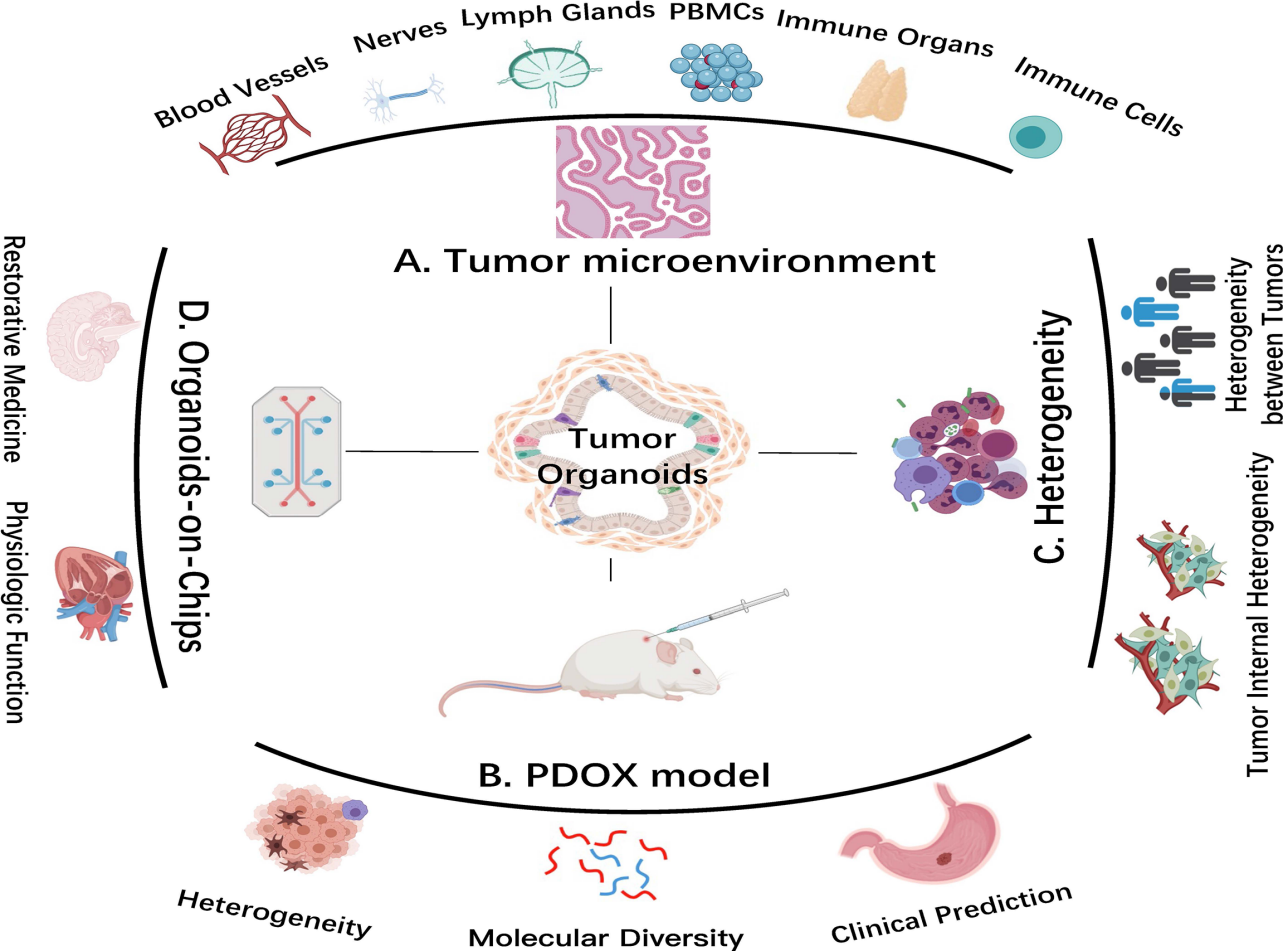
Advanced organoid technology in cancer research. **(A)** Coculture system of tumor organoids with components of the tumor microenvironment (TME). **(B)** Patient-derived organoid xenograft (PDOX) model. The PDOX model indicates tumor heterogeneity and molecular diversity and predicts clinical treatment *in vivo*. **(C)** Tumor organoids better reflect tumor heterogeneity. **(D)** Organoids-on-Chips system. This system combines organoid and tissue engineering to highly replicate physiological function *in vitro* and to facilitate regenerative medical development. Images are adapted from Servier Medical Art (https://smart.servier.com).

The inherent self-organizing of stem cells does not signify that organoid might form fine tissue under any condition (41). This process emphasizes that fate guides organoid to develop into mimic-tissues in a highly environment-dependent manner (42). Those established organoid-forming approaches have considerable defects: when cultured for too long, stem cells would uncontrollably develop into a circular cystic closed structure, with a short life span and non-physiological shape, resulting in inconsistency between organoid and organs in anatomy and physiology (43, 44). To solve this issue, bioengineering cultivated organoids into a variety of biomaterials that can promote their better proliferation, precise differentiation, and exact function (43, 45, 46). Tissue bioengineering uses bioactive substances to regenerate or repair tissues through *in vitro* construction (**Figure 2**) (47). It is accomplished by controlling the process of organoids and establishing the next generation with high physiological correlation (48). Researchers at the EPFL Institute have constructed an intestinal geometric scaffold with hydrogel, providing an appropriate place to guide organoid to form a true intestinal organ (49). In this method, stem cells were cultured in scaffolds simulating the surface of natural tissue and then combined into microfluidic chips. Due to their inherent self-organization, organoids grew on tubular scaffolds and self-organized to form intestines (47). Organoids would gradually form continuous cell layers with recess structure and villous-like domain and form “mini-intestines” *in vitro*, which maintain the same functional features as primary organs *in vivo (* 48, 50, 51). Improved methods have been used for organoid generation (i.e., stomach, liver, kidney, etc.) (52–55).

Microfluidic approach and organoid chip integrate mechanical and physiological parameters, expanding the usage of organoids for recapitulating the physiological function of their source organs (**Figure 2**) (37). Using organ setting on a chip, a single gastric organoid has been successfully established (56). Not singly but in pairs, the use of lumen flow and pressure circulation to induce peristaltic movement showed the feasibility of integrating engineering in organoid culture (57). Coincidentally, kidney organoids on chips were exposed to shear stress by applying fluid flow, thereby mimicking the kidney environment *in vivo (* 58). The existence of fluid flow not only promoted maturation of renal organoids but also was conducive to formation of vascular network with perfusion lumen. Creative combination of organoids and tissue engineering allows organoid growth, which may overcome space constraints and promote shape-guided morphogenesis and physiology. Furthermore, researchers used micro fabricated cell rejection microporous (an apparatus for culturing organoids using tissue engineering) to culture and monitor homogeneous liver organoids from dissociated human iPSCs (54). Organoid bioengineering improves experimental replication, optimizes model quality, and realizes clinical transformation. Decembrini et al. (59) have proven that production of retinal organoids was accelerated and standardized in the best physicochemical microenvironment. A biomimetic hydrogel composed of circular bottom microwell arrays and optimized media formulation not only facilitated mouse ESC formation but also aggregated retinal organoids in a stereotypical manner, leading to an unlimited source of retinal neurons (59). Combining organoid culture with tissue engineering, organ devices accurately control organoid growth, while organoids simulate real physiology more in line with the situation *in vivo* (60–62).

## Organoid as a Model for Tumorigenesis

Compared with traditional two-dimensional culture of tumor cells, tumor organoids maintain better cell heterogeneity, retain tumor characteristics, show less loss of tumor niche components, and provide a more authentic environment for clinical treatment (63, 64). In contrast to patient-derived tumor xenografts (PDXs), the success rate of tumor organoid construction is way higher (50%–90% vs. 10%–30%), organoids are maintained for a longer time with lower cost, so it is easy to gene editing and large-scale drug screening (65–68). There are two kinds of organoid construction techniques, one is derived from differentiation of iPSCs (16) and the other is directly derived from tumor tissues (69). Constructing tumor organoids from iPSCs largely depends on tumor types, and the culture operation is more complex (70). Tumor organoids obtained by iPSC differentiation lose the complexity of the tumor microenvironment (TME) (71). The more common organoid culture method is to directly use primary tissues, supplemented by cytokines, tumor matrix, and other components (70, 72). Tumor organoids such as colorectal, breast, pancreatic, prostate, liver, and gastric cancers have been successfully constructed (73–77). Researchers have obtained a diverse collection of tumor organoids with different characteristics through *in vitro* culture to constitute a living organoid biobank (78). Through histochemical observation of the morphology of tumor organoids, their internal structure was like that of primary tissue (1, 4, 6). Simultaneously, genome and single-cell sequencing was carried out to explore the variation between organoids and primary tumors in the gene mutation spectrum (79).

Organoids maintain tumor heterogeneity within and among tumors (80). Patient-derived organoids (PDOs) maintains the diversity and complexity of tumor origin in terms of cell hetereogeniety, histology, gene mutation, transcription spectrum, and even metabolism (39, 81, 82). Organoids not only reflect characteristics of primary tumors, but also exert advantages for explorations of tumorigenesis, cell communication, epigenetics, and invasion (34, 77).

Due to various sources of tumor samples and slightly discrepant culture system among laboratories, it is not easy to duplicate the experimental findings, and this hinders further verification and reference of obtained data. A study conducted by Dr. Manel Esteller, director of the Josep Carreras leukemia Institute (IJC), used epigenetic Infinium MethylationEPIC BeadChip (EPIC), a microarray chip from Illumina that interrogates more than 850,000 CpG sites to analyze DNA methylation status of 25 human cancer organoids (83). This data indicated that tumor organoids highly sustain biological properties and heterogeneity of tumor tissue *in situ*.

### Tumorigenesis in Organoids

Tumor originates from accumulation of gene mutation in normal somatic cells; while not all mutations have access to induce tumorigenesis, tolerance of different tissues to the same mutation is widely divergent. Numerous cell and animal experiments have clarified key factors and decisive mechanisms initiated from gene mutation to tumorigenesis, fully understanding that such process is artificial due to the failure of monitoring and intervening the earliest process of tumor development. Innovative organoid system makes it possible to understand the transformation process from normal tissue to tumor (84). The Clustered Regularly Interspaced Short Palindromic Repeats (CRISPR)/Cas9 system has revolutionized genetic engineering, allowing gene editing of normal organoids by inducing oncogenes to obtain tumor organoids and track the carcinogenic process from initial to advanced stage (85–87). Mutated tumor-related genes *KRAS*, *CDKN2A*, *TP53*, and *SMAD4* in normal pancreatic organoids eventually developed to a state recapitulating pancreatic ductal adenocarcinoma (80, 88, 89). Lannagan et al. (90) found that mutated intestinal organoids could grow and reproduce without relying on any exogenous cytokines. When transplanting organoids with four mutations into mice, these organoids induced by colorectal cancer were invasive (90). This approach can amplify the effects of driving mutations in the same genetic background. Organoids from normal tissues mimic tumor pathogenesis by continuously introducing cancer-driven mutations (91, 92). Combination of organoid system and CRISPR/Cas9 strategy is powerful to study the origin of tumor (93, 94).

It is generally believed that cancer is a progressive disease caused by accumulation of abnormal gene mutation. These genetic anomalies include tumor suppressor/oncogene mutations and chromosome abnormalities (95). It is increasingly clear that tumors can be induced by epigenetic changes. Epigenetics refers to heritable change of gene function without a change of genetic material, eventually leading to different phenotypes (96). It mainly includes DNA methylation, histone modification, non-coding RNA regulation, and chromatin structure reconstruction. Potential tumor suppressor genes are inhibited or silenced at the transcriptional level by DNA methylation, promoting malignant transformation from normal cells (97, 98). It has begun to dig influence and underlying mechanisms that modulate methylation and chromatin states of tumor suppressor gene or tumor oncogene during tumor formation (96, 99). Epigenetic changes bring abundant precancerous cell expansion. They first occur in precancerous cells, determining subsequent genetic changes that promote malignant transformation and tumor cell clonal expansion (98, 99). Aloia et al. (100) have demonstrated that bile duct cells underwent epigenetic modification of genome-wide DNA methylation during tissue damage and subsequent organoid construction. This work that was inspired by those epigenetic changes of organoids from normal to malignant cell transition was able to determine subsequent uncontrollable modification of genetic machinery, eventually leading to malignant occurrence and development (95, 98, 100).

### Tumor Microenvironment in Organoid System

TME is an internal condition for the production and survival of tumor cells, including immune and inflammatory cells, fibroblasts, vascular endothelial cells (101). Bidirectional communication between tumor cells and TME possesses an indispensable position in tumor promotion (36). Tumor-infiltrating lymphocytes (TILs) are heterogeneously composed of different lymphocytes; their phenotypic and functional characteristics largely correlate with interaction with tumor cells (102). Researchers cultured tumor organoids through air–liquid interface to reproduce TME *in vitro*. Air-PDOs successfully retained inherent fibrous matrix and a variety of immune cell components of primary tumor tissue (103). Through integrated culture, the *in situ* tumor essence and matrix were reserved, including functional TILs. By continuously coculturing cancer organoids and peripheral blood monocytes (PBMCs) in the presence of T cell-stimulating growth factors, antigen-specific cytotoxic T cells were selected and amplified in about half of total samples (**Figure 2**). T cells expanded from adjacent healthy epithelial tissues resulted in undisturbed organoid expansion without a significant level of organoid cytotoxicity (104).

Cancer-associated fibroblasts (CAFs) secrete miscellaneous cytokines, chemokines, and growth factors to create a conducive microenvironment for tumor progression (105). The prominent role of CAFs is considered to shape stem cell niche to cultivate cancer stem cells (CSCs), while two-dimensional culture of cell lines is far from satisfactory in summarizing general characteristics of CSCs (106). The first coculture model of CAFs and organoids was established in pancreatic cancer, followed by in liver, colorectal, prostate, esophageal, and breast cancers (107–110). Recently, it is reported that organoids and CAFs from diethylnitrosamine (DEN)-induced mouse liver tumors can be cultured together; CAFs promoted organoid growth through paracrine signals. Cotransplantation of CAFs with liver tumor organoids promoted tumor growth in xenotransplantation models; CAFs may not regulate the efficiency of organoid initiation but accelerated its growth (111).

The deficiency of blood vessels in the organoid system brings challenges (112). One study emphasized that endothelial cells can be replaced by adaptable angiogenic cells to form a perfusion plastic vascular plexus for restrictive synthetic semipermeable membrane by organ chip system, and this provided a physiological platform for tumor organoid vascularization (113). This minimized hypoxia in tumor organoids provided a ponder over influence and mechanism of vascular endothelial cells on tumor occurrence and development (**Figure 2**) (112, 114).

## Application of Organoids in Precision Tumor Oncotherapy

Tumors are generally heterogeneous, and there is no fixed treatment for all types of tumors, which makes precision medicine a new direction for tumor therapy, that is, patients with different stages of tumor are entitled to discrepant treatment schemes (115, 116). Tumor organoids replicate complex signaling pathways and cell-to-cell relationship and more accurately reflect tumor genetic features (79). Organoids become a personalized treatment channel with its unique experimental advantages—high level of physiological relevance and convenience of *in vitro* operation (4, 65, 117).

### Application of Organoids in Tumor Medication

Organoid technology for precision medicine refers to drug screening *in vitro* in PDOs and formulation of individual medication (32, 85). Tumor organoids largely maintain heterogeneity between source tumors and different patients, individual morphology and scale of organoids remain such uniform (3, 38, 118). These organoids not only remove confusing variables that may be introduced from animal models but also provide greater complexity than homogenized cell cultures (1, 44, 66). The valuable tumor model established by patients’ iPSCs is able to understand tumor pathogenesis and disease progression (3, 9). The established living biobank of tumor organoids has infinite predictive value for determining the distinct drug response of patients. In turn, it gives access to large-scale drug screening (63, 119). Tumor organoids swiftly select the optimal therapeutic schedule for patients, accompanied by reducing side effects and tumor recurrence (24, 70, 120). They significantly shorten the preclinical test period, working as a key slot in drug discovery, providing a large amount of biological data and a high-quality platform (6, 33). With the progress and standardization of organoid culture, the accessibility of tumor organs will be more widely realized (25, 30, 121).

Tumor organoid-based drug screening integrated with next-generation sequencing (NGS) is conducive to oncotherapy, which is combined with clinical treatment to form complementarity (122–124). NGS detects genetic mutations of patients at source and provides drug treatment options, but it alone does not guarantee clinical efficacy (125–128). As a distinct supplement, organoid is advantageous to well investigate this uncertainty (6, 65, 117). Patients with epidermal growth factor receptor amplification were usually treated with cetuximab guided by NGS, yet this consequence was overturned by the organoid system, which was consistent with actual clinical situation (129). Organoid drug screening further picks more effective approaches on the basis of sequencing to give conclusive recommendations and practical biology evidence for patients (20). This technology clarifies particular therapeutics for precision medicine and determines whether a particular group of patients is not suitable for a therapeutic (3, 70).

### Application of Organoids in Tumor Vaccination

**Figure 3:**
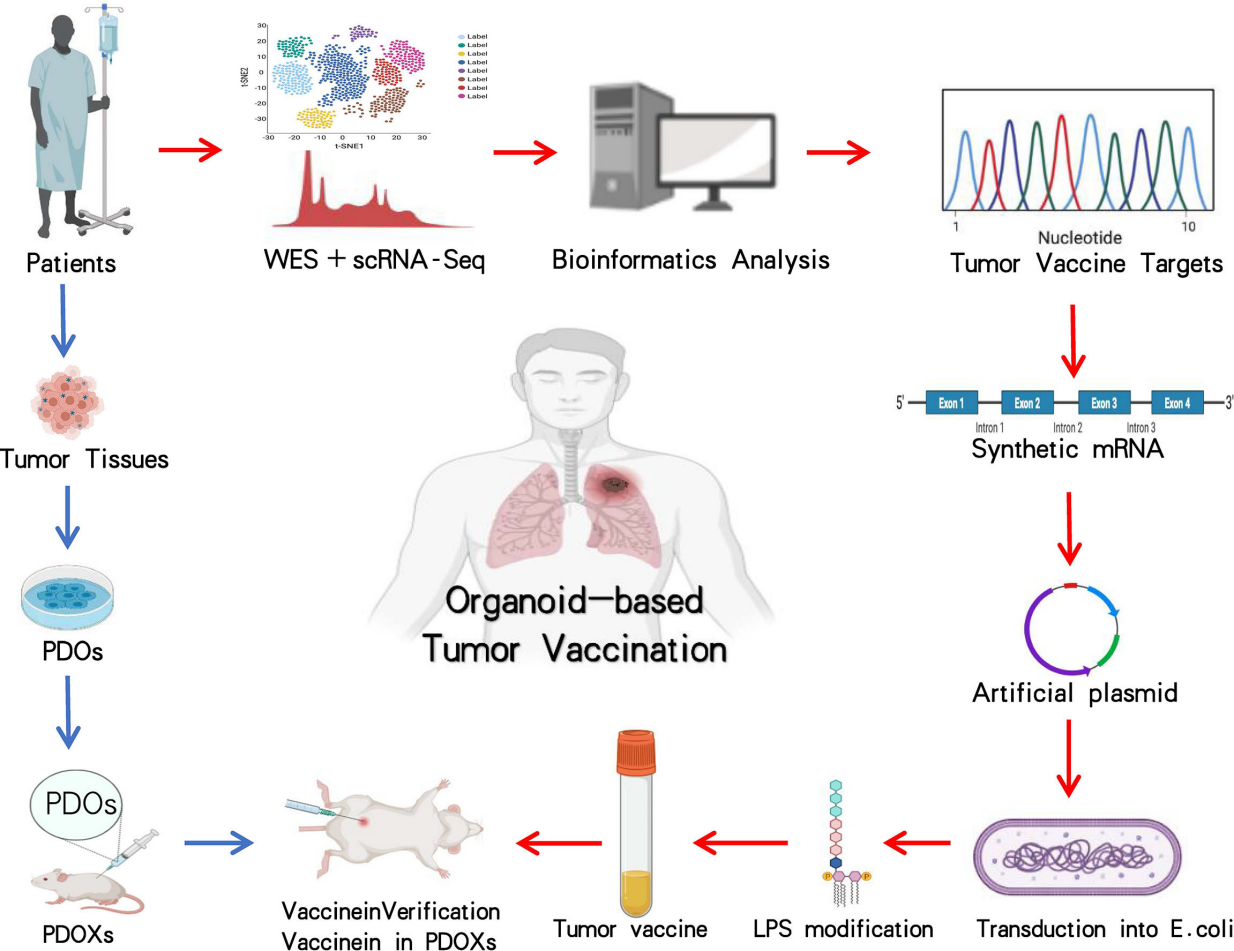
Application and investigation of organoid-based tumor vaccination. The workflow with blue arrows represents the patient-derived organoid xenograft (PDOX) model, and processes with red arrows indicate how tumor vaccine is generated. The entire steps are to verify the efficacy of tumor vaccination by using the PDOX model. Images are adapted from Servier Medical Art (https://smart.servier.com).

Organoid culture along with NGS and single-cell sequencing (scRNA-seq) is good to hunt for therapeutic targets and discover mutation-associated neoantigens (MANAs) for fresh targeted remedy or tumor vaccination (**Figure 3**) (130–134). With continuous innovation and improvement of benchwork for appraising tumor MANAs, such as the recently emerging NeoScreen technology, more previously hidden tumor antigen epitopes have been identified (135–138). Based on bioinformatics data analysis, MANAs reflecting individual disease are unearthed by a machine learning algorithm (138, 139). Using an organoid system, Demmers et al. (140) recently considered that human leukocyte antigen (HLA) class I peptide expression among different clonal cells from the same colorectal cancer patient was variability and its widespread difference in cloning specificity was generally common. By linking organoid proteomics and HLA peptide ligandomics, they discovered that tumor-specific ligands derived from DNA damage and tumor suppressor proteins were remarkably presented in tumor cells, which might be consistent with defunction of their cytoprotective effect. In general, their data demonstrated heterogeneous HLA peptide expression in an individual patient and presumed that a promising multipeptide tumor vaccine may be a feasible option to minimize immune escape risk.

Taking certain mRNAs encoding MANAs as available templates, individualized tumor vaccines against MANAs are artificially synthesized, stimulating tumor-specific T-cell production for the sake of diminishing tumor cells (141–147). These tumor vaccines are theoretically proven to be potential therapeutics for accurate oncotherapy (146, 148, 149). Organoids have been implanted during the development and testing process of vaccines against pathogenic microorganisms including bacteria and viruses (141, 150–152). Researchers developed mini tonsils *in vitro* from surgery tissues and added coronavirus disease 2019 (COVID-19) candidate vaccines into the culture system, a predominant tool to verify vaccine efficacy, to observe whether tonsil organoids incite immune responses, specific immunocytes, and antibodies against viral surface spike proteins (153). It is believed that in the near future, organoids might be exploited in research and development of tumor vaccines (140, 142, 147, 149). PDOs with target gene mutations and autologous or non-autologous immune cells are cocultured. The constructed tumor vaccine candidates are subjected to the coculture system to detect tumor-specific T-cell production and tumor vaccine (145, 148, 154, 155). This emerging platform would be applied to the development of tumor vaccines on a large scale (**Figure 3**) (3, 36, 37, 65). Tumor organoids provide attractive options for tumor vaccine and are expected to be a unique utensil for tumor precision medicine (146, 153, 156).

### Application of Organoids in Tumor Chemoprevention and Nanomedicine

Tumor chemoprevention generally refers to inhibition of tumor and vascular cell proliferation, which is expected to maintain its efficacy through the whole stage of chemotherapy for patients (157). It is required to develop advanced approaches for better tumor chemoprevention. The organoid model, a three-dimensional culture platform for primary cells, paves the way of evolution for tumor chemoprevention and provides a practical tool for tumor treatment. A study demonstrated that an A Disintegrin and Metalloprotease 10 inhibitor prevented glioma stem cells from integrating into brain organoids, similar to the mouse xenotransplantation outcome. Temozolomide and Adriamycin treatment reduced the size of tumor organoids by about 30% and 80%, respectively, but had no effect on normal nerve cell organoids. It is worth evaluating the reactivity of glioblastoma multiforme tumor and healthy brain cells when exposed to therapeutics by using the organoid system. They confirmed the biological correlation between organoids and clinical data, providing a basis for high-throughput drug screening and determining the most effective drug timely (158). One additional study indicated that the combination of extracellular signal-regulated kinase 1/2 (ERK1/2) inhibition and autophagy process reduced liver metastases in mice with pancreatic ductal adenocarcinoma PDO transplantation. Over the past decade, vitamin D_3_ has aroused great interest as a chemoprophylactic agent, especially for treating neoplasms from the digestive system (159). A recent comparative study reported that PDOs revealed the homeostatic effect of vitamin D_3_ on human intestinal mucosa (160). The organoid technique offers a promising preclinical model for evaluating chemotherapeutic efficacy, a more in-depth mechanistic insight into tumor biology and an organoid biobank system for exploring optimal tumor chemoprevention (161).

Current challenges of organoid application in the clinics include low reproducibility and presence of mixed cell populations, limiting strict experimental examination (38, 42, 77). We have described that the combination of biomaterials and organoids may address the abovementioned deficiencies. Astrocytes and neurons were generated in depart, and they were combined with microplates in the desired proportion and size. This approach accelerated maturation of astrocytes and allowed chemical or genetic manipulation of any cell type before coculture (162, 163). However, how to select certain types of cells as targets for transgenic or epigenetic manipulation, drug delivery, or local extracellular modification is still a bench-side issue (158, 159). Nanoparticles generated from polymer or liposomes are utilized as encapsulation carriers for astrocytes and other cells (164). In another innovative application of nanomedicine, iPSC-derived neural cells and the astrocyte neuron coculture system have been applied as a screening platform to evaluate neurotoxicity. Toxicity is a major risk for nanomedicine; the safety of any nanoparticles needs to be tested prior to application *in vivo* (165). An alternative way to reduce toxicity is to use exosomes as a source of nanocarriers, which can increase cell type-specific targeting and enhance their functionality by engineering surface antibodies. Verification of the toxicity of nanomaterials in organoids requires much further attention (158, 159, 164).

## Challenges and Prospects of Organoids

Organoid technology has made breakthroughs in regenerative medicine and tumor biology. Organoids have a wide range of potential applications, while they still face technical drawbacks (117, 118). Most of the existing organoids are derived from epithelial cells; non-epithelial-originated organoids are hard to be established and maintained for a long purpose (i.e., primary glioblastoma) (121). Coculture of tumor organoids with immune cells is a predictable model for cell therapy, but the culture condition requires further optimization. Different tumor types and individual patients maintain heterogeneity; it is difficult to accurately simulate about its dynamic adaptation (81). The classical organoid culture system contains non-human animal products, such as ECM and hydrogel (Matrigel or BME); these may bring undiscovered effects to the organoid biology. Further organoid-bioengineering strategy and conditioned medium developed with artificial substrates may solve the abovementioned limitations (166, 167). The organoid technology potentiates more accurate tumor recapitulation than cell lines and the PDX model (20, 168). The organoid culture system is good to predict drug sensitivity, tumor promotion, and tumor vaccination (119, 169). Having the *in vivo* modeling accessibility and developing application, organoid technology is expected to have a profound impact on both bench and bedside for precision medicine (84, 120).

## Author Contributions

ZW, SZ, and XL wrote the draft. ZL XX, and AH reviewed the article. GC and JK collected data. ZM and YW provided study materials. DX was responsible for review conception, design, and revision. All authors have read and agreed to the published version of the article.

## Funding

This research was funded by the Natural Science Foundation of China (82173358 to AH and 32170924 to DX) and the Shanghai Municipal Committee of Science and Technology (21140901600 to DX).

## Conflict of Interest

ZM and YW served as lab technicians for Shanghai OneTar Biomedicine Co. Ltd.

The remaining authors declare that the research was conducted in the absence of any commercial or financial relationships that could be construed as a potential conflict of interest.

## Publisher’s Note

All claims expressed in this article are solely those of the authors and do not necessarily represent those of their affiliated organizations, or those of the publisher, the editors and the reviewers. Any product that may be evaluated in this article, or claim that may be made by its manufacturer, is not guaranteed or endorsed by the publisher.

## References

[1] Artegiani B Clevers H. Use and Application of 3D-Organoid Technology. Hum Mol Genet (2018) 27:R99–R107. doi: 10.1093/hmg/ddy187 29796608

[2] McCusker D. Cellular Self-Organization: Generating Order From the Abyss. Mol Biol Cell (2020) 31:143–8. doi: 10.1091/mbc.E19-04-0207 PMC7001480 31999511

[3] Pasch CA Favreau PF Yueh AE Babiarz CP Gillette AA Sharick JT. Patient-Derived Cancer Organoid Cultures to Predict Sensitivity to Chemotherapy and Radiation. Clin Cancer Res (2019) 25:5376–87. doi: 10.1158/1078-0432.CCR-18-3590 PMC6726566 31175091

[4] Clevers HC. Organoids: Avatars for Personalized Medicine. Keio J Med (2019) 68(4):95. doi: 10.2302/kjm.68-006-ABST 31875622

[5] Liu H Zhang Y Zhang YY Li YP Hua ZQ Zhang CJ. Human Embryonic Stem Cell-Derived Organoid Retinoblastoma Reveals a Cancerous Origin. Proc Natl Acad Sci USA (2020) 117:33628–38. doi: 10.1073/pnas.2011780117 PMC7776986 33318192

[6] Clevers H. Modeling Development and Disease With Organoids. Cell (2016) 165:1586–97. doi: 10.1016/j.cell.2016.05.082 27315476

[7] Bershteyn M Kriegstein AR. Cerebral Organoids in a Dish: Progress and Prospects. Cell (2013) 155:19–20. doi: 10.1016/j.cell.2013.09.010 24074857 PMC5127703

[8] Karagiannis P Takahashi K Saito M Yoshida Y Okita K Watanabe A. Induced Pluripotent Stem Cells and Their Use in Human Models of Disease and Development. Physiol Rev (2019) 99:79–114. doi: 10.1152/physrev.00039.2017 30328784

[9] Ohnuki M Takahashi K. Present and Future Challenges of Induced Pluripotent Stem Cells. Philos Trans R Soc Lond B Biol Sci (2015) 370:20140367. doi: 10.1098/rstb.2014.0367 26416678 PMC4633996

[10] Lim WF Inoue-Yokoo T Tan KS Lai MI Sugiyama D. Hematopoietic Cell Differentiation From Embryonic and Induced Pluripotent Stem Cells. Stem Cell Res Ther (2013) 4:71. doi: 10.1186/scrt222 23796405 PMC3706875

[11] Blokzijl F de Ligt J Jager M Sasselli V Roerink S Sasaki N. Tissue-Specific Mutation Accumulation in Human Adult Stem Cells During Life. Nature (2016) 538:260–4. doi: 10.1038/nature19768 PMC5536223 27698416

[12] Huch M Bonfanti P Boj SF Sato T Loomans CJ van de Wetering M. Unlimited *In Vitro* Expansion of Adult Bi-Potent Pancreas Progenitors Through the Lgr5/R-Spondin Axis. EMBO J (2013) 32:2708–21. doi: 10.1038/emboj.2013.204 PMC3801438 24045232

[13] Sato T Vries RG Snippert HJ van de Wetering M Barker N Stange DE. Single Lgr5 Stem Cells Build Crypt-Villus Structures *In Vitro* Without a Mesenchymal Niche. Nature (2009) 459:262–5. doi: 10.1038/nature07935 19329995

[14] Ootani A Li X Sangiorgi E Ho QT Ueno H Toda S. Sustained *In Vitro* Intestinal Epithelial Culture Within a Wnt-Dependent Stem Cell Niche. Nat Med (2009) 15:701–6. doi: 10.1038/nm.1951 PMC2919216 19398967

[15] Fujii M Matano M Toshimitsu K Takano A Mikami Y Nishikori S. Human Intestinal Organoids Maintain Self-Renewal Capacity and Cellular Diversity in Niche-Inspired Culture Condition. Cell Stem Cell (2018) 23:787–93.e786. doi: 10.1016/j.stem.2018.11.016 30526881

[16] Spence JR Mayhew CN Rankin SA Kuhar MF Vallance JE Tolle K. Directed Differentiation of Human Pluripotent Stem Cells Into Intestinal Tissue In Vitro. Nature (2011) 470:105–9. doi: 10.1038/nature09691 PMC3033971 21151107

[17] Fan YY Davidson LA Chapkin RS. Murine Colonic Organoid Culture System and Downstream Assay Applications. Methods Mol Biol (2019) 1576:171–81. doi: 10.1007/7651_2016_8 PMC5316509 27539462

[18] Muzio L Consalez GG. Modeling Human Brain Development With Cerebral Organoids. Stem Cell Res Ther (2013) 4(6):154. doi: 10.1186/scrt384 24367992 PMC4055082

[19] Elbadawy M Usui T Mori T Tsunedomi R Hazama S Nabeta R. Establishment of a Novel Experimental Model for Muscle-Invasive Bladder Cancer Using a Dog Bladder Cancer Organoid Culture. Cancer Sci (2019) 110:2806–21. doi: 10.1111/cas.14118 PMC6726682 31254429

[20] Dedhia PH Bertaux-Skeirik N Zavros Y Spence JR. Organoid Models of Human Gastrointestinal Development and Disease. Gastroenterology (2016) 150:1098–112. doi: 10.1053/j.gastro.2015.12.042 PMC4842135 26774180

[21] Sato T Stange DE Ferrante M Vries RG Van Es JH Van den Brink S. Long-Term Expansion of Epithelial Organoids From Human Colon, Adenoma, Adenocarcinoma, and Barrett's Epithelium. Gastroenterology (2011) 141:1762–72. doi: 10.1053/j.gastro.2011.07.050 21889923

[22] Fujii M Sato T. Somatic Cell-Derived Organoids as Prototypes of Human Epithelial Tissues and Diseases. Nat Mater (2021) 20:156–69. doi: 10.1038/s41563-020-0754-0 32807924

[23] Date S Sato T. Mini-Gut Organoids: Reconstitution of the Stem Cell Niche. Annu Rev Cell Dev Biol (2015) 31:269–89. doi: 10.1146/annurev-cellbio-100814-125218 26436704

[24] Wang J Li X Chen H. Organoid Models in Lung Regeneration and Cancer. Cancer Lett (2020) 475:129–35. doi: 10.1016/j.canlet.2020.01.030 32032677

[25] Drost J Karthaus WR Gao D Driehuis E Sawyers CL Chen Y. Organoid Culture Systems for Prostate Epithelial and Cancer Tissue. Nat Protoc (2016) 11:347–58. doi: 10.1038/nprot.2016.006 PMC4793718 26797458

[26] Yousef Yengej FA Jansen J Rookmaaker MB Verhaar MC Clevers H. Kidney Organoids and Tubuloids. Cells (2020) 9(6):1326. doi: 10.3390/cells9061326 PMC7349753 32466429

[27] Sachs N Papaspyropoulos A Zomer-van Ommen DD Heo I Bottinger L Klay D. Long-Term Expanding Human Airway Organoids for Disease Modeling. EMBO J (2019) 38(4):e100300. doi: 10.15252/embj.2018100300 30643021 PMC6376275

[28] Lancaster MA Knoblich JA. Organogenesis in a Dish: Modeling Development and Disease Using Organoid Technologies. Science (2014) 345:1247125. doi: 10.1126/science.1247125 25035496

[29] Lancaster MA Renner M Martin CA Wenzel D Bicknell LS Hurles ME. Cerebral Organoids Model Human Brain Development and Microcephaly. Nature (2013) 501:373–9. doi: 10.1038/nature12517 PMC3817409 23995685

[30] Dutta D Heo I Clevers H. Disease Modeling in Stem Cell-Derived 3d Organoid Systems. Trends Mol Med (2017) 23:393–410. doi: 10.1016/j.molmed.2017.02.007 28341301

[31] Nanki K Toshimitsu K Takano A Fujii M Shimokawa M Ohta Y. Divergent Routes Toward Wnt and R-Spondin Niche Independency During Human Gastric Carcinogenesis. Cell (2018) 174:856–69.e817. doi: 10.1016/j.cell.2018.07.027 30096312

[32] Huch M Knoblich JA Lutolf MP Martinez-Arias A. The Hope and the Hype of Organoid Research. Development (2017) 144:938–41. doi: 10.1242/dev.150201 28292837

[33] Turco MY Gardner L Hughes J Cindrova-Davies T Gomez MJ Farrell L. Long-Term, Hormone-Responsive Organoid Cultures of Human Endometrium in a Chemically Defined Medium. Nat Cell Biol (2017) 19:568–77. doi: 10.1038/ncb3516 PMC5410172 28394884

[34] Seino T Kawasaki S Shimokawa M Tamagawa H Toshimitsu K Fujii M. Human Pancreatic Tumor Organoids Reveal Loss of Stem Cell Niche Factor Dependence During Disease Progression. Cell Stem Cell (2018) 22:454–67.e456. doi: 10.1016/j.stem.2017.12.009 29337182

[35] Gabriel E Albanna W Pasquini G Ramani A Josipovic N Mariappan A. Human Brain Organoids Assemble Functionally Integrated Bilateral Optic Vesicles. Cell Stem Cell (2021) 28:1740–57.e1748. doi: 10.1016/j.stem.2021.07.010 34407456

[36] Bar-Ephraim YE Kretzschmar K Clevers H. Organoids in Immunological Research. Nat Rev Immunol (2020) 20:279–93. doi: 10.1038/s41577-019-0248-y 31853049

[37] Sun W Luo Z Lee J Kim HJ Lee K Tebon P. Organ-On-a-Chip for Cancer and Immune Organs Modeling. Adv Healthc Mater (2019) 8:e1801363. doi: 10.1002/adhm.201801363 30605261 PMC6424124

[38] Rossi G Manfrin A Lutolf MP. Progress and Potential in Organoid Research. Nat Rev Genet (2018) 19:671–87. doi: 10.1038/s41576-018-0051-9 30228295

[39] Xu H Lyu X Yi M Zhao W Song Y Wu K. Organoid Technology and Applications in Cancer Research. J Hematol Oncol (2018) 11:116. doi: 10.1186/s13045-018-0662-9 30219074 PMC6139148

[40] Bannier-Helaouet M Post Y Korving J Trani Bustos M Gehart H Begthel H. Exploring the Human Lacrimal Gland Using Organoids and Single-Cell Sequencing. Cell Stem Cell (2021) 28:1221–32.e1227. doi: 10.1016/j.stem.2021.02.024 33730555

[41] Florian S Iwamoto Y Coughlin M Weissleder R Mitchison TJ. A Human Organoid System That Self-Organizes to Recapitulate Growth and Differentiation of a Benign Mammary Tumor. Proc Natl Acad Sci USA (2019) 116:11444–53. doi: 10.1073/pnas.1702372116 PMC6561274 31101720

[42] Semertzidou A Brosens JJ McNeish I Kyrgiou M. Organoid Models in Gynaecological Oncology Research. Cancer Treat Rev (2020) 90:102103. doi: 10.1016/j.ctrv.2020.102103 32932156

[43] Yin X Mead BE Safaee H Langer R Karp JM Levy O. Engineering Stem Cell Organoids. Cell Stem Cell (2016) 18:25–38. doi: 10.1016/j.stem.2015.12.005 26748754 PMC4728053

[44] Saengwimol D Rojanaporn D Chaitankar V Chittavanich P Aroonroch R Boontawon T. Three-Dimensional Organoid Model Recapitulates Tumorigenic Aspects and Drug Responses of Advanced Human Retinoblastoma. Sci Rep (2018) 8:15664. doi: 10.1038/s41598-018-34037-y 30353124 PMC6199308

[45] Takebe T Wells JM. Organoids by Design. Science (2019) 364(6444):956–9. doi: 10.1126/science.aaw7567 PMC8212787 31171692

[46] Cruz-Acuna R Quiros M Farkas AE Dedhia PH Huang S Siuda D. Synthetic Hydrogels for Human Intestinal Organoid Generation and Colonic Wound Repair. Nat Cell Biol (2017) 19:1326–35. doi: 10.1038/ncb3632 PMC5664213 29058719

[47] Grimm D Egli M Kruger M Riwaldt S Corydon TJ Kopp S. Tissue Engineering Under Microgravity Conditions-Use of Stem Cells and Specialized Cells. Stem Cells Dev (2018) 27:787–804. doi: 10.1089/scd.2017.0242 29596037

[48] Rahmani S Breyner NM Su HM Verdu EF Didar TF. Intestinal Organoids: A New Paradigm for Engineering Intestinal Epithelium In Vitro. Biomaterials (2019) 194:195–214. doi: 10.1016/j.biomaterials.2018.12.006 30612006

[49] Nikolaev M Mitrofanova O Broguiere N Geraldo S Dutta D Tabata Y. Homeostatic Mini-Intestines Through Scaffold-Guided Organoid Morphogenesis. Nature (2020) 585:574–8. doi: 10.1038/s41586-020-2724-8 32939089

[50] Sato T Clevers H. Growing Self-Organizing Mini-Guts From a Single Intestinal Stem Cell: Mechanism and Applications. Science (2013) 340(6137):1190–4. doi: 10.1126/science.1234852 23744940

[51] Li VSW. Modelling Intestinal Inflammation and Infection Using 'Mini-Gut' Organoids. Nat Rev Gastroenterol Hepatol (2021) 18:89–90. doi: 10.1038/s41575-020-00391-4 33257835 PMC7703732

[52] Mahe MM Aihara E Schumacher MA Zavros Y Montrose MH Helmrath MA. Establishment of Gastrointestinal Epithelial Organoids. Curr Protoc Mouse Biol (2013) 3:217–40. doi: 10.1002/9780470942390.mo130179 PMC4120977 25105065

[53] Li X Nadauld L Ootani A Corney DC Pai RK Gevaert O. Oncogenic Transformation of Diverse Gastrointestinal Tissues in Primary Organoid Culture. Nat Med (2014) 20:769–77. doi: 10.1038/nm.3585 PMC4087144 24859528

[54] Liu H Wang Y Cui K Guo Y Zhang X Qin J. Advances in Hydrogels in Organoids and Organs-On-a-Chip. Adv Mater (2019) 31:e1902042. doi: 10.1002/adma.201902042 31282047

[55] Mitaka T Ooe H. Characterization of Hepatic-Organoid Cultures. Drug Metab Rev (2010) 42:472–81. doi: 10.3109/03602530903492020 20025558

[56] Park SE Georgescu A Huh D. Organoids-On-a-Chip. Science (2019) 364(6444):960–5. doi: 10.1126/science.aaw7894 PMC7764943 31171693

[57] Sugihara K Yamaguchi Y Usui S Nashimoto Y Hanada S Kiyokawa E. A New Perfusion Culture Method With a Self-Organized Capillary Network. PLoS One (2020) 15:e0240552. doi: 10.1371/journal.pone.0240552 33112918 PMC7592787

[58] Homan KA Gupta N Kroll KT Kolesky DB Skylar-Scott M Miyoshi T. Flow-Enhanced Vascularization and Maturation of Kidney Organoids In Vitro. Nat Methods (2019) 16:255–62. doi: 10.1038/s41592-019-0325-y PMC6488032 30742039

[59] Decembrini S Hoehnel S Brandenberg N Arsenijevic Y Lutolf MP. Hydrogel-Based Milliwell Arrays for Standardized and Scalable Retinal Organoid Cultures. Sci Rep (2020) 10:10275. doi: 10.1038/s41598-020-67012-7 32581233 PMC7314858

[60] He J Zhang X Xia X Han M Li F Li C. Organoid Technology for Tissue Engineering. J Mol Cell Biol (2020) 12:569–79. doi: 10.1093/jmcb/mjaa012 PMC7683016 32249317

[61] Tan Q Choi KM Sicard D Tschumperlin DJ. Human Airway Organoid Engineering as a Step Toward Lung Regeneration and Disease Modeling. Biomaterials (2017) 113:118–32. doi: 10.1016/j.biomaterials.2016.10.046 PMC5121055 27815996

[62] Garreta E Kamm RD Chuva de Sousa Lopes SM Lancaster MA Weiss R Trepat X. Rethinking Organoid Technology Through Bioengineering. Nat Mater (2021) 20:145–55. doi: 10.1038/s41563-020-00804-4 33199860

[63] Tuveson D Clevers H. Cancer Modeling Meets Human Organoid Technology. Science (2019) 364(6444):952–5. doi: 10.1126/science.aaw6985 31171691

[64] Ji DB Wu AW. Organoid in Colorectal Cancer: Progress and Challenges. Chin Med J (Engl) (2020) 133:1971–7. doi: 10.1097/CM9.0000000000000882 PMC7462208 32826461

[65] Aboulkheyr Es H Montazeri L Aref AR Vosough M Baharvand H. Personalized Cancer Medicine: An Organoid Approach. Trends Biotechnol (2018) 36:358–71. doi: 10.1016/j.tibtech.2017.12.005 29366522

[66] Bleijs M van de Wetering M Clevers H Drost J. Xenograft and Organoid Model Systems in Cancer Research. EMBO J (2019) 38:e101654. doi: 10.15252/embj.2019101654 31282586 PMC6670015

[67] Rowe RG Daley GQ. Induced Pluripotent Stem Cells in Disease Modelling and Drug Discovery. Nat Rev Genet (2019) 20:377–88. doi: 10.1038/s41576-019-0100-z PMC6584039 30737492

[68] Shinozawa T Kimura M Cai Y Saiki N Yoneyama Y Ouchi R. High-Fidelity Drug-Induced Liver Injury Screen Using Human Pluripotent Stem Cell-Derived Organoids. Gastroenterology (2021) 160:831–46.e810. doi: 10.1053/j.gastro.2020.10.002 33039464 PMC7878295

[69] Maru Y Hippo Y. Current Status of Patient-Derived Ovarian Cancer Models. Cells (2019) 8(5):505. doi: 10.3390/cells8050505 PMC6562658 31130643

[70] Wang Z Wang SN Xu TY Miao ZW Su DF Miao CY. Organoid Technology for Brain and Therapeutics Research. CNS Neurosci Ther (2017) 23:771–8. doi: 10.1111/cns.12754 PMC6492716 28884977

[71] Smith RC Tabar V. Constructing and Deconstructing Cancers Using Human Pluripotent Stem Cells and Organoids. Cell Stem Cell (2019) 24:12–24. doi: 10.1016/j.stem.2018.11.012 30581078 PMC6516073

[72] Fujii M Shimokawa M Date S Takano A Matano M Nanki K. A Colorectal Tumor Organoid Library Demonstrates Progressive Loss of Niche Factor Requirements During Tumorigenesis. Cell Stem Cell (2016) 18:827–38. doi: 10.1016/j.stem.2016.04.003 27212702

[73] Xie BY Wu AW. Organoid Culture of Isolated Cells From Patient-Derived Tissues With Colorectal Cancer. Chin Med J (Engl) (2016) 129:2469–75. doi: 10.4103/0366-6999.191782 PMC5072260 27748340

[74] Baker LA Tiriac H Clevers H Tuveson DA. Modeling Pancreatic Cancer With Organoids. Trends Cancer (2016) 2:176–90. doi: 10.1016/j.trecan.2016.03.004 PMC4847151 27135056

[75] Jain T Dudeja V. The War Against Pancreatic Cancer in 2020 - Advances on All Fronts. Nat Rev Gastroenterol Hepatol (2021) 18:99–100. doi: 10.1038/s41575-020-00410-4 33414515

[76] Xu AT Tong JL Ran ZH. Organoids Derived From Digestive Tract, Liver, and Pancreas. J Dig Dis (2016) 17:3–10. doi: 10.1111/1751-2980.12305 26666830

[77] Seidlitz T Merker SR Rothe A Zakrzewski F von Neubeck C Grutzmann K. Human Gastric Cancer Modelling Using Organoids. Gut (2019) 68:207–17. doi: 10.1136/gutjnl-2017-314549 PMC6352409 29703791

[78] Kawasaki K Toshimitsu K Matano M Fujita M Fujii M Togasaki K. An Organoid Biobank of Neuroendocrine Neoplasms Enables Genotype-Phenotype Mapping. Cell (2020) 183:1420–35.e1421. doi: 10.1016/j.cell.2020.10.023 33159857

[79] Driehuis E van Hoeck A Moore K Kolders S Francies HE Gulersonmez MC. Pancreatic Cancer Organoids Recapitulate Disease and Allow Personalized Drug Screening. Proc Natl Acad Sci USA (2019) 116(52):26580–90. doi: 10.1073/pnas.1911273116 PMC6936689 31818951

[80] Hu X Zhang L Li Y Ma X Dai W Gao X. Organoid Modelling Identifies That DACH1 Functions as a Tumour Promoter in Colorectal Cancer by Modulating BMP Signalling. EBioMedicine (2020) 56:102800. doi: 10.1016/j.ebiom.2020.102800 32512510 PMC7281795

[81] Huang B Trujillo MA Fujikura K Qiu M Chen F Felsenstein M. Molecular Characterization of Organoids Derived From Pancreatic Intraductal Papillary Mucinous Neoplasms. J Pathol (2020) 252:252–62. doi: 10.1002/path.5515 PMC8162794 32696980

[82] Jin RU Mills JC. Tumor Organoids to Study Gastroesophageal Cancer: A Primer. J Mol Cell Biol (2020) 12:593–606. doi: 10.1093/jmcb/mjaa035 32652008 PMC7683018

[83] Joshi R Castro De Moura M Pineyro D Alvarez-Errico D Arribas C Esteller M. The DNA Methylation Landscape of Human Cancer Organoids Available at the American Type Culture Collection. Epigenetics (2020) 15:1167–77. doi: 10.1080/15592294.2020.1762398 PMC7595613 32396494

[84] Kretzschmar K. Cancer Research Using Organoid Technology. J Mol Med (Berl) (2021) 99:501–15. doi: 10.1007/s00109-020-01990-z PMC8026469 33057820

[85] Fan H Demirci U Chen P. Emerging Organoid Models: Leaping Forward in Cancer Research. J Hematol Oncol (2019) 12:142. doi: 10.1186/s13045-019-0832-4 31884964 PMC6936115

[86] Artegiani B Hendriks D Beumer J Kok R Zheng X Joore I. Fast and Efficient Generation of Knock-in Human Organoids Using Homology-Independent CRISPR-Cas9 Precision Genome Editing. Nat Cell Biol (2020) 22:321–31. doi: 10.1038/s41556-020-0472-5 32123335

[87] Hendriks D Artegiani B Hu H Chuva de Sousa Lopes S Clevers H. Establishment of Human Fetal Hepatocyte Organoids and CRISPR-Cas9-Based Gene Knockin and Knockout in Organoid Cultures From Human Liver. Nat Protoc (2021) 16:182–217. doi: 10.1038/s41596-020-00411-2 33247284

[88] Paes Dias M Tripathi V van der Heijden I Cong K Manolika EM Bhin J. Loss of Nuclear DNA Ligase III Reverts PARP Inhibitor Resistance in BRCA1/53BP1 Double-Deficient Cells by Exposing ssDNA Gaps. Mol Cell (2021) 81(22):4692–708.e9. doi: 10.1016/j.molcel.2021.09.005 PMC9098260 34555355

[89] Nguyen AT Lee SY Chin HJ Le QV Lee D. Kinase Activity of ERBB3 Contributes to Intestinal Organoids Growth and Intestinal Tumorigenesis. Cancer Sci (2020) 111:137–47. doi: 10.1111/cas.14235 PMC6942447 31724799

[90] Lannagan TRM Lee YK Wang T Roper J Bettington ML Fennell L. Genetic Editing of Colonic Organoids Provides a Molecularly Distinct and Orthotopic Preclinical Model of Serrated Carcinogenesis. Gut (2019) 68:684–92. doi: 10.1136/gutjnl-2017-315920 PMC6192855 29666172

[91] Artegiani B van Voorthuijsen L Lindeboom RGH Seinstra D Heo I Tapia P. Probing the Tumor Suppressor Function of BAP1 in CRISPR-Engineered Human Liver Organoids. Cell Stem Cell (2019) 24:927–43.e926. doi: 10.1016/j.stem.2019.04.017 31130514

[92] Ballabio C Anderle M Gianesello M Lago C Miele E Cardano M. Modeling Medulloblastoma *In Vivo* and With Human Cerebellar Organoids. Nat Commun (2020) 11:583. doi: 10.1038/s41467-019-13989-3 31996670 PMC6989674

[93] Li H Dai W Xia X Wang R Zhao J Han L. Modeling Tumor Development and Metastasis Using Paired Organoids Derived From Patients With Colorectal Cancer Liver Metastases. J Hematol Oncol (2020) 13:119. doi: 10.1186/s13045-020-00957-4 32883331 PMC7650218

[94] Takeda H Kataoka S Nakayama M Ali MAE Oshima H Yamamoto D. CRISPR-Cas9-Mediated Gene Knockout in Intestinal Tumor Organoids Provides Functional Validation for Colorectal Cancer Driver Genes. Proc Natl Acad Sci USA (2019) 116:15635–44. doi: 10.1073/pnas.1904714116 PMC6681705 31300537

[95] Hanahan D. Hallmarks of Cancer: New Dimensions. Cancer Discov (2022) 12:31–46. doi: 10.1158/2159-8290.CD-21-1059 35022204

[96] Stener-Victorin E Deng Q. Epigenetic Inheritance of Polycystic Ovary Syndrome - Challenges and Opportunities for Treatment. Nat Rev Endocrinol (2021) 17:521–33. doi: 10.1038/s41574-021-00517-x 34234312

[97] Jeffries MA. The Development of Epigenetics in the Study of Disease Pathogenesis. Adv Exp Med Biol (2020) 1253:57–94. doi: 10.1007/978-981-15-3449-2_2 32445091

[98] Ushijima T Clark SJ Tan P. Mapping Genomic and Epigenomic Evolution in Cancer Ecosystems. Science (2021) 373(6562):1474–9. doi: 10.1126/science.abh1645 34554797

[99] Carter B Zhao K. The Epigenetic Basis of Cellular Heterogeneity. Nat Rev Genet (2021) 22:235–50. doi: 10.1038/s41576-020-00300-0 PMC10880028 33244170

[100] Aloia L McKie MA Vernaz G Cordero-Espinoza L Aleksieva N van den Ameele J. Epigenetic Remodelling Licences Adult Cholangiocytes for Organoid Formation and Liver Regeneration. Nat Cell Biol (2019) 21:1321–33. doi: 10.1038/s41556-019-0402-6 PMC6940196 31685987

[101] Koliaraki V Prados A Armaka M Kollias G. The Mesenchymal Context in Inflammation, Immunity and Cancer. Nat Immunol (2020) 21:974–82. doi: 10.1038/s41590-020-0741-2 32747813

[102] Lin B Du L Li H Zhu X Cui L Li X. Tumor-Infiltrating Lymphocytes: Warriors Fight Against Tumors Powerfully. BioMed Pharmacother (2020) 132:110873. doi: 10.1016/j.biopha.2020.110873 33068926

[103] Neal JT Li X Zhu J Giangarra V Grzeskowiak CL Ju J. Organoid Modeling of the Tumor Immune Microenvironment. Cell (2018) 175:1972–88.e1916. doi: 10.1016/j.cell.2018.11.021 30550791 PMC6656687

[104] Dijkstra KK Cattaneo CM Weeber F Chalabi M van de Haar J Fanchi LF. Generation of Tumor-Reactive T Cells by Co-Culture of Peripheral Blood Lymphocytes and Tumor Organoids. Cell (2018) 174:1586–98.e1512. doi: 10.1016/j.cell.2018.07.009 30100188 PMC6558289

[105] Nurmik M Ullmann P Rodriguez F Haan S Letellier E. In Search of Definitions: Cancer-Associated Fibroblasts and Their Markers. Int J Cancer (2020) 146:895–905. doi: 10.1002/ijc.32193 30734283 PMC6972582

[106] Sahai E Astsaturov I Cukierman E DeNardo DG Egeblad M Evans RM. A Framework for Advancing Our Understanding of Cancer-Associated Fibroblasts. Nat Rev Cancer (2020) 20:174–86. doi: 10.1038/s41568-019-0238-1 PMC7046529 31980749

[107] Tsai S McOlash L Palen K Johnson B Duris C Yang Q. Development of Primary Human Pancreatic Cancer Organoids, Matched Stromal and Immune Cells and 3D Tumor Microenvironment Models. BMC Cancer (2018) 18:335. doi: 10.1186/s12885-018-4238-4 29587663 PMC5870823

[108] Chakrabarti J Koh V So JBY Yong WP Zavros YA. Preclinical Human-Derived Autologous Gastric Cancer Organoid/Immune Cell Co-Culture Model to Predict the Efficacy of Targeted Therapies. J Vis Exp (2021) 173:e61443. doi: 10.3791/61443 34309588

[109] Naruse M Ochiai M Sekine S Taniguchi H Yoshida T Ichikawa H. Re-Expression of REG Family and DUOXs Genes in CRC Organoids by Co-Culturing With CAFs. Sci Rep (2021) 11:2077. doi: 10.1038/s41598-021-81475-2 33483567 PMC7822883

[110] Xu R Zhou X Wang S Trinkle C. Tumor Organoid Models in Precision Medicine and Investigating Cancer-Stromal Interactions. Pharmacol Ther (2021) 218:107668. doi: 10.1016/j.pharmthera.2020.107668 32853629 PMC7855432

[111] Liu J Li P Wang L Li M Ge Z Noordam L. Cancer-Associated Fibroblasts Provide a Stromal Niche for Liver Cancer Organoids That Confers Trophic Effects and Therapy Resistance. Cell Mol Gastroenterol Hepatol (2021) 11:407–31. doi: 10.1016/j.jcmgh.2020.09.003 PMC7788239 32932015

[112] Palikuqi B Nguyen DT Li G Schreiner R Pellegata AF Liu Y. Adaptable Haemodynamic Endothelial Cells for Organogenesis and Tumorigenesis. Nature (2020) 585:426–32. doi: 10.1038/s41586-020-2712-z PMC7480005 32908310

[113] Skardal A Murphy SV Devarasetty M Mead I Kang HW Seol YJ. Multi-Tissue Interactions in an Integrated Three-Tissue Organ-on-a-Chip Platform. Sci Rep (2017) 7:8837. doi: 10.1038/s41598-017-08879-x 28821762 PMC5562747

[114] Cakir B Xiang Y Tanaka Y Kural MH Parent M Kang YJ. Engineering of Human Brain Organoids With a Functional Vascular-Like System. Nat Methods (2019) 16:1169–75. doi: 10.1038/s41592-019-0586-5 PMC6918722 31591580

[115] Weil AR. Precision Medicine. Health Aff (Millwood) (2018) 37:687. doi: 10.1377/hlthaff.2018.0520 29733714

[116] Dagogo-Jack I Shaw AT. Tumour Heterogeneity and Resistance to Cancer Therapies. Nat Rev Clin Oncol (2018) 15:81–94. doi: 10.1038/nrclinonc.2017.166 29115304

[117] Li Y Tang P Cai S Peng J Hua G. Organoid Based Personalized Medicine: From Bench to Bedside. Cell Regener (2020) 9:21. doi: 10.1186/s13619-020-00059-z PMC7603915 33135109

[118] The Promise of Organoids and Embryoids. Nat Mater (2021) 20:121. doi: 10.1038/s41563-021-00926-3 33504988

[119] Abugomaa A Elbadawy M Yamawaki H Usui T Sasaki K. Emerging Roles of Cancer Stem Cells in Bladder Cancer Progression, Tumorigenesis, and Resistance to Chemotherapy: A Potential Therapeutic Target for Bladder Cancer. Cells (2020) 9(1):235. doi: 10.3390/cells9010235 PMC7016964 31963556

[120] Fatehullah A Tan SH Barker N. Organoids as an *In Vitro* Model of Human Development and Disease. Nat Cell Biol (2016) 18:246–54. doi: 10.1038/ncb3312 26911908

[121] Andreatta F Beccaceci G Fortuna N Celotti M De Felice D Lorenzoni M. The Organoid Era Permits the Development of New Applications to Study Glioblastoma. Cancers (Basel) (2020) 12(11):3303. doi: 10.3390/cancers12113303 PMC7695252 33182346

[122] Narasimhan V Wright JA Churchill M Wang T Rosati R Lannagan TRM. Medium-Throughput Drug Screening of Patient-Derived Organoids From Colorectal Peritoneal Metastases to Direct Personalized Therapy. Clin Cancer Res (2020) 26:3662–70. doi: 10.1158/1078-0432.CCR-20-0073 PMC8366292 32376656

[123] Krall N Superti-Furga G Vladimer GI. Patient-Derived Model Systems and the Development of Next-Generation Anticancer Therapeutics. Curr Opin Chem Biol (2020) 56:72–8. doi: 10.1016/j.cbpa.2020.01.002 32086157

[124] Aberle MR Burkhart RA Tiriac H Olde Damink SWM Dejong CHC Tuveson DA. Patient-Derived Organoid Models Help Define Personalized Management of Gastrointestinal Cancer. Br J Surg (2018) 105:e48–60. doi: 10.1002/bjs.10726 PMC5774241 29341164

[125] Morganti S Tarantino P Ferraro E D'Amico P Viale G Trapani D. Complexity of Genome Sequencing and Reporting: Next Generation Sequencing (NGS) Technologies and Implementation of Precision Medicine in Real Life. Crit Rev Oncol Hematol (2019) 133:171–82. doi: 10.1016/j.critrevonc.2018.11.008 30661654

[126] Yang F Anekpuritanang T Press RD. Clinical Utility of Next-Generation Sequencing in Acute Myeloid Leukemia. Mol Diagn Ther (2020) 24:1–13. doi: 10.1007/s40291-019-00443-9 31848884

[127] Doostparast Torshizi A Wang K. Next-Generation Sequencing in Drug Development: Target Identification and Genetically Stratified Clinical Trials. Drug Discovery Today (2018) 23:1776–83. doi: 10.1016/j.drudis.2018.05.015 29758342

[128] Tripathi P Singh J Lal JA Tripathi V. Next-Generation Sequencing: An Emerging Tool for Drug Designing. Curr Pharm Des (2019) 25:3350–7. doi: 10.2174/1381612825666190911155508 31544713

[129] Vlachogiannis G Hedayat S Vatsiou A Jamin Y Fernandez-Mateos J Khan K. Patient-Derived Organoids Model Treatment Response of Metastatic Gastrointestinal Cancers. Science (2018) 359:920–6. doi: 10.1126/science.aao2774 PMC6112415 29472484

[130] Skora AD Douglass J Hwang MS Tam AJ Blosser RL Gabelli SB. Generation of MANAbodies Specific to HLA-Restricted Epitopes Encoded by Somatically Mutated Genes. Proc Natl Acad Sci USA (2015) 112:9967–72. doi: 10.1073/pnas.1511996112 PMC4538619 26216968

[131] Danilova L Anagnostou V Caushi JX Sidhom JW Guo H Chan HY. The Mutation-Associated Neoantigen Functional Expansion of Specific T Cells (MANAFEST) Assay: A Sensitive Platform for Monitoring Antitumor Immunity. Cancer Immunol Res (2018) 6:888–99. doi: 10.1158/2326-6066.CIR-18-0129 PMC6072595 29895573

[132] Alcazer V Bonaventura P Tonon L Wittmann S Caux C Depil S. Neoepitopes-Based Vaccines: Challenges and Perspectives. Eur J Cancer (2019) 108:55–60. doi: 10.1016/j.ejca.2018.12.011 30648630

[133] Zhang W Yin Q Huang H Lu J Qin H Chen S. Personal Neoantigens From Patients With NSCLC Induce Efficient Antitumor Responses. Front Oncol (2021) 11:628456:628456. doi: 10.3389/fonc.2021.628456 33928024 PMC8076796

[134] Caushi JX Zhang J Ji Z Vaghasia A Zhang B Hsiue EH. Transcriptional Programs of Neoantigen-Specific TIL in Anti-PD-1-Treated Lung Cancers. Nature (2021) 596:126–32. doi: 10.1038/s41586-021-03752-4 PMC8338555 34290408

[135] Arnaud M Chiffelle J Genolet R Navarro Rodrigo B Perez MAS Huber F. Sensitive Identification of Neoantigens and Cognate TCRs in Human Solid Tumors. Nat Biotechnol (2021). doi: 10.1038/s41587-021-01072-6 PMC9110298 34782741

[136] Peng M Mo Y Wang Y Wu P Zhang Y Xiong F. Neoantigen Vaccine: An Emerging Tumor Immunotherapy. Mol Cancer (2019) 18(1):128. doi: 10.1186/s12943-019-1055-6 31443694 PMC6708248

[137] Gopanenko AV Kosobokova EN Kosorukov VS. Main Strategies for the Identification of Neoantigens. Cancers (Basel) (2020) 12(10):2879. doi: 10.3390/cancers12102879 PMC7600129 33036391

[138] Roudko V Greenbaum B Bhardwaj N. Computational Prediction and Validation of Tumor-Associated Neoantigens. Front Immunol (2020) 11:27:27. doi: 10.3389/fimmu.2020.00027 32117226 PMC7025577

[139] Richters MM Xia H Campbell KM Gillanders WE Griffith OL Griffith M. Best Practices for Bioinformatic Characterization of Neoantigens for Clinical Utility. Genome Med (2019) 11:56. doi: 10.1186/s13073-019-0666-2 31462330 PMC6714459

[140] Demmers LC Kretzschmar K Van Hoeck A Bar-Epraïm YE van den Toorn HWP Koomen M. Single-Cell Derived Tumor Organoids Display Diversity in HLA Class I Peptide Presentation. Nat Commun (2020) 11(1):5338. doi: 10.1038/s41467-020-19142-9 33087703 PMC7577990

[141] Roth C Cantaert T Colas C Prot M Casademont I Levillayer L. A Modified mRNA Vaccine Targeting Immunodominant NS Epitopes Protects Against Dengue Virus Infection in HLA Class I Transgenic Mice. Front Immunol (2019) 10:1424:1424. doi: 10.3389/fimmu.2019.01424 31293584 PMC6598640

[142] Wang Y Zhang L Xu Z Miao L Huang L. mRNA Vaccine With Antigen-Specific Checkpoint Blockade Induces an Enhanced Immune Response Against Established Melanoma. Mol Ther (2018) 26(2):420–34. doi: 10.1016/j.ymthe.2017.11.009 PMC5835019 29249397

[143] Keskin DB Anandappa AJ Sun J Tirosh I Mathewson ND Li S. Neoantigen Vaccine Generates Intratumoral T Cell Responses in Phase Ib Glioblastoma Trial. Nature (2019) 565:234–9. doi: 10.1038/s41586-018-0792-9 PMC6546179 30568305

[144] Hu Z Leet DE Allesøe RL Oliveira G Li S Luoma AM. Personal Neoantigen Vaccines Induce Persistent Memory T Cell Responses and Epitope Spreading in Patients With Melanoma. Nat Med (2021) 27(3):515–25. doi: 10.1038/s41591-020-01206-4 PMC8273876 33479501

[145] Li L Goedegebuure SP Gillanders WE. Preclinical and Clinical Development of Neoantigen Vaccines. Ann Oncol (2017) 28(suppl_12):xii11–7. doi: 10.1093/annonc/mdx681 PMC5834106 29253113

[146] Blass E Ott PA. Advances in the Development of Personalized Neoantigen-Based Therapeutic Cancer Vaccines. Nat Rev Clin Oncol (2021) 18(4):215–29. doi: 10.1038/s41571-020-00460-2 PMC7816749 33473220

[147] Stanton SE Gad E Corulli LR Lu H Disis ML. Tumor-Associated Antigens Identified Early in Mouse Mammary Tumor Development Can Be Effective Vaccine Targets. Vaccine (2019) 37:3552–61. doi: 10.1016/j.vaccine.2019.05.024 PMC7087478 31126858

[148] Castle JC Kreiter S Diekmann J Lower M van de Roemer N de Graaf J. Exploiting the Mutanome for Tumor Vaccination. Cancer Res (2012) 72:1081–91. doi: 10.1158/0008-5472.CAN-11-3722 22237626

[149] Song Q Zhang CD Wu XH. Therapeutic Cancer Vaccines: From Initial Findings to Prospects. Immunol Lett (2018) 196:11–21. doi: 10.1016/j.imlet.2018.01.011 29407608

[150] Marrazzo P Cricca M Nastasi C. Are the Organoid Models an Invaluable Contribution to ZIKA Virus Research? Pathogens (2021) 10(10):1233. doi: 10.3390/pathogens10101233 34684182 PMC8537471

[151] Liu X Mondal AM. Conditional Cell Reprogramming for Modeling Host-Virus Interactions and Human Viral Diseases. J Med Virol (2020) 92:2440–52. doi: 10.1002/jmv.26093 PMC7586785 32478897

[152] Ahammad I Lira SS. Designing a Novel mRNA Vaccine Against SARS-CoV-2: An Immunoinformatics Approach. Int J Biol Macromol (2020) 162:820–37. doi: 10.1016/j.ijbiomac.2020.06.213 PMC7319648 32599237

[153] Wagar LE Salahudeen A Constantz CM Wendel BS Lyons MM Mallajosyula V. Modeling Human Adaptive Immune Responses With Tonsil Organoids. Nat Med (2021) 27:125–35. doi: 10.1038/s41591-020-01145-0 PMC7891554 33432170

[154] Pulendran B Davis MM. The Science and Medicine of Human Immunology. Science (2020) 369(6511):eaay4014. doi: 10.1126/science.aay4014 32973003 PMC7872131

[155] Wisnewski AV Redlich CA Liu J Kamath K Abad QA Smith RF. Immunogenic Amino Acid Motifs and Linear Epitopes of COVID-19 mRNA Vaccines. PLoS One (2021) 16:e0252849. doi: 10.1371/journal.pone.0252849 34499652 PMC8428655

[156] Lu L Jiang J Zhan M Zhang H Wang QT Sun SN. Targeting Tumor-Associated Antigens in Hepatocellular Carcinoma for Immunotherapy: Past Pitfalls and Future Strategies. Hepatology (2021) 73:821–32. doi: 10.1002/hep.31502 32767586

[157] Walczak K Marciniak S Rajtar G. Cancer Chemoprevention - Selected Molecular Mechanisms. Postepy Hig Med Dosw (Online) (2017) 71(0):149–61. doi: 10.5604/01.3001.0010.3799 28258675

[158] Scuto M Ontario ML Salinaro AT Caligiuri I Rampulla F Zimbone V. Redox Modulation by Plant Polyphenols Targeting Vitagenes for Chemoprevention and Therapy: Relevance to Novel Anti-Cancer Interventions and Mini-Brain Organoid Technology. Free Radic Biol Med (2022) 179:59–75. doi: 10.1016/j.freeradbiomed.2021.12.267 34929315

[159] Scuto M Trovato Salinaro A Caligiuri I Ontario ML Greco V Sciuto N. Redox Modulation of Vitagenes *via* Plant Polyphenols and Vitamin D: Novel Insights for Chemoprevention and Therapeutic Interventions Based on Organoid Technology. Mech Ageing Dev (2021) 199:111551. doi: 10.1016/j.mad.2021.111551 34358533

[160] Umezawa S Higurashi T Komiya Y Arimoto J Horita N Kaneko T. Chemoprevention of Colorectal Cancer: Past, Present, and Future. Cancer Sci (2019) 110:3018–26. doi: 10.1111/cas.14149 PMC6778640 31361372

[161] Gairola K Gururani S Bahuguna A Garia V Pujari R Dubey SK. Natural Products Targeting Cancer Stem Cells: Implications for Cancer Chemoprevention and Therapeutics. J Food Biochem (2021) 45:e13772. doi: 10.1111/jfbc.13772 34028051

[162] Rickner H Jiang L Hong R Wolozin B Cheng C. Single Cell Transcriptomic Profiling of Neurodegeneration Mediated by Tau in a Novel 3D Neuron-Astrocyte Coculture Model. Alzheimers Dement (2021) 17 Suppl 2:e058551. doi: 10.1002/alz.058551

[163] Flannagan K Stopperan JA Troutwine BM Lysaker CR Strope T Draper J. Mitochondrial Phenotypes in iPSC AD Models. Alzheimers Dement (2021) 17(Suppl 2):e058489. doi: 10.1002/alz.058489

[164] Wang S Wang D Duan Y Zhou Z Gao W Zhang L. Cellular Nanosponges for Biological Neutralization. Adv Mater (2021) e2107719. doi: 10.1002/adma.202107719 34783078

[165] Yang K Yang Z Yu G Nie Z Wang R Chen X. Polyprodrug Nanomedicines: An Emerging Paradigm for Cancer Therapy. Adv Mater (2021) 34(6):e2107434. doi: 10.1002/adma.202107434 34693571

[166] Giobbe GG Crowley C Luni C Campinoti S Khedr M Kretzschmar K. Extracellular Matrix Hydrogel Derived From Decellularized Tissues Enables Endodermal Organoid Culture. Nat Commun (2019) 10:5658. doi: 10.1038/s41467-019-13605-4 31827102 PMC6906306

[167] Gjorevski N Lutolf MP. Synthesis and Characterization of Well-Defined Hydrogel Matrices and Their Application to Intestinal Stem Cell and Organoid Culture. Nat Protoc (2017) 12:2263–74. doi: 10.1038/nprot.2017.095 28981121

[168] O'Rourke KP Loizou E Livshits G Schatoff EM Baslan T Manchado E. Transplantation of Engineered Organoids Enables Rapid Generation of Metastatic Mouse Models of Colorectal Cancer. Nat Biotechnol (2017) 35:577–82. doi: 10.1038/nbt.3837 PMC5462850 28459450

[169] Ooft SN Weeber F McLean CM Kaing S van Werkhoven E Dijkstra KK. Patient-Derived Organoids Can Predict Response to Chemotherapy in Metastatic Colorectal Cancer Patients. Sci Transl Med (2019) 11(513):eaay2574. doi: 10.1126/scitranslmed.aay2574 31597751

